# Oxyresveratrol Inhibits TNF-α-Stimulated Cell Proliferation in Human Immortalized Keratinocytes (HaCaT) by Suppressing AKT Activation

**DOI:** 10.3390/pharmaceutics14010063

**Published:** 2021-12-28

**Authors:** Nitwara Wikan, Phateep Hankittichai, Phatarawat Thaklaewphan, Saranyapin Potikanond, Wutigri Nimlamool

**Affiliations:** 1 Department of Pharmacology, Faculty of Medicine, Chiang Mai University, Chiang Mai 50200, Thailand; nitwara.wik@cmu.ac.th (N.W.); phateep.han18@gmail.com (P.H.); phatarawat.th@gmail.com (P.T.); saranyapin.p@cmu.ac.th (S.P.); 2 Research Center for Development of Local Lanna Rice and Rice Products, Chiang Mai University, Chiang Mai 50200, Thailand

**Keywords:** psoriasis, PI3K, AKT, keratinocyte, HaCaT cells, proliferation, TNF-α, oxyresveratrol

## Abstract

Psoriasis is a complex inflammatory disease characterized by hyperproliferative keratinocyte caused by active PI3K/AKT signaling. TNF-α concentrated in the psoriatic lesions stimulates AKT activation. We previously discovered that oxyresveratrol inhibited inflammation via suppressing AKT phosphorylation, therefore oxyresveratrol may possess a conserved property to block AKT activation and proliferation in keratinocyte in response to TNF-α. Our current study proved that oxyresveratrol exhibited potent anti-proliferative effects against TNF-α. These effects are explained by the findings that oxyresveratrol could potentially inhibit TNF-α-stimulated AKT and GSK3-_β_ activation in a dose-dependent manner, and its inhibitory pattern was comparable to that of a specific PI3K inhibitor. Results from immunofluorescence supported that oxyresveratrol effectively inhibited AKT and GSK3-_β_ activation in individual cells upon TNF-α stimulation. Furthermore, functional assay confirmed that oxyresveratrol repressed the expansion of the HaCaT colony over 3 days, and this was caused by the ability of oxyresveratrol to induce cell cycle arrest at S and G2/M phases and the reduction in the expression of a proliferative marker (Ki-67) and a survival marker (MCL-1). Given the importance of TNF-α and the PI3K/AKT pathway in the psoriatic phenotype, we anticipate that oxyresveratrol, which targets the TNF-α-stimulated PI3K/AKT pathway, would represent a promising psoriasis therapy in the near future.

## 1. Introduction

Psoriasis is well established to be an incurable chronic inflammatory dermatosis that impacts people around the globe [1,2]. The obvious characteristic of psoriasis is erythema scalelike skin plaques which are contributed from epidermal keratinocyte hyperplasia, aberrant differentiation, parakeratosis, and chronic dermis inflammation [3]. Currently, there is no radical cure for psoriasis since this condition is complex and is considered to be a chronic inflammatory skin disease. One of its complications is that the disease activity is linked to the level of inflammatory cytokines. Tumor necrosis factor-alpha (TNF-α) is well defined to be a major factor which regulates several genes responsible for immune and inflammatory responses [4]. TNF-α is secreted by T lymphocytes and plays a regulatory role in the pathogenic process during the development of psoriasis [5]. Although the abnormality of T-lymphocytes in psoriatic lesions and the inflammatory role of TNF-α have been associated with the pathogenesis of psoriasis, the cause of excessive keratinocyte proliferation is not completely understood [6]. Considering the degree of the mode of action, TNF-α is a potent pro-inflammatory cytokine that plays a crucial role in diverse physiological and pathological processes [7]. In normal cells, TNF-α has been presented as involved with the regeneration process by governing cytokine production of immune cells during the inflammation step [8] and promoting cell proliferation via increasing growth factor secretion [9]. However, there are numerous reports revealing that unusual function or exaggerated TNF-α releasing participates in various disease occurrence [10]. Raised TNF-α level in the microenvironment of ovarian cancer was observed and it was expected that it was considerably related with tumor cell growth [11]. In addition, TNF-α is believed to be one of the key mediators causing chronic psoriasis because it can trigger constitutive inflammatory cytokine releasing, including IL-17 and IL-23, and lead to uncontrollable cell proliferation [12]. A high TNF-α amount in tissue lesions was found in most patients [13]. TNF-α is a significant inflammatory cytokine that has been well defined to generate a concert of cytokines relevant to the pathogenesis of psoriasis [14]. It has been reported that an increased level of TNF-α was detected in the serum of patients with psoriasis, and its rising was associated with disease activity [15]. In addition to the aspect of its inflammatory aggravation, TNF-α can stimulate the proliferation of normal healthy cells [16,17]. This cytokine also plays a major role in promoting the initiation and progression of cancer cells [18]. In particular, TNF-α potently induces pancreatic cancer metastasis in a mouse model which can be effectively inhibited by infliximab or etanercept (TNF-α inhibitors) [19]. Additionally, TNF-α has been reported to promote proliferation of several different solid tumors including ovarian, intestinal, and skin tumors [20,21]. The effect of TNF-α on cell proliferation may be regulated in part through the ability of this cytokine to activate PI3K/AKT signal transduction pathway. The PI3K/AKT signal transduction pathway is responsible for a variety of important physiologic cellular functions which include inhibition of apoptosis, cell survival, cell cycle progression, cell proliferation, and angiogenesis [22,23,24,25]. Accumulated evidence emphasizes the association of the PI3K/AKT pathway with clinical relevance in inflammatory diseases, including psoriasis [26,27,28]. With this reason, inhibition of the PI3K/AKT signaling pathway could be developed to be a novel way to treat psoriasis.

Accumulated studies have revealed that proliferation of human keratinocytes is dependent on the PI3K/AKT signaling pathway [29]. Many studies have reported the aberrance of several elements of the PI3K/AKT signaling cascade in patients with psoriasis. One significant discovery demonstrated that PI3K is specifically overexpressed in psoriatic lesions in comparison with that in lesions of chronic dermatitis, seborrheic keratosis, squamous cell carcinoma, and basal cell carcinoma [30]. Additional research has provided information that phosphorylation of AKT is dominantly increased in the keratinocyte in psoriasis lesions in comparison with normal and non-lesional skin [31,32]. On the basis that the PI3K/AKT pathway is abnormally stimulated in hyperproliferation keratinocyte, this key signal transduction pathway has emerged as a therapeutic target.

Over the past decade, it has been reported that some small molecules specifically inhibiting the PI3K/AKT signaling provide great outcomes for psoriatic arthritis, and this may be an effective management for psoriasis [33]. Studies in animal models demonstrated that some PI3K/AKT inhibitors could prevent development of IMQ-induced psoriasis in mice [34,35]. Moreover, clinical data have suggested that PI3K/AKT inhibitors may improve therapeutic benefit for psoriasis [36]. Therefore, the discovery and development of novel agents that can inhibit hyperactivity of the PI3K/AKT may help improve treatment effectiveness for psoriasis.

Oxyresveratrol (*trans*-2,3′,4,5′-tetrahydroxystilbene, OXY) is a natural stilbene that has lately gained prominence due to its simple chemical composition and varied pharmacological properties [37]. Particularly for its anti-inflammatory activities, OXY inhibited the lipopolysaccharide (LPS)-mediated release of nitric oxide (NO), IL-6, MCP-1, and TNF-α in RAW 264.7 macrophages and IL-6 and MCP-1 secretion in HMC3 human microglial cells induced by IL-1β by suppressing PI3K/AKT phosphorylation [38,39]. Moreover, keratinocytes trigger an inflammatory process through the stimulation of TNF-α [40,41]. TNF receptor activation through the NF-κB and PI3K/AKT pathways causes an increase in the production of various inflammatory cytokines and chemokines that promote cell proliferation and cell survival, leading to immune response [42,43]. As a step towards the development of PI3K/AKT-based treatment, we focused on this interesting natural compound since it possesses strong potency to suppress AKT activation stimulated by a key psoriatic cytokine. We believe that oxyresveratrol is a good candidate to be developed as a promising agent targeting hyperactive PI3K/AKT pathway for the psoriasis therapy in the near future.

## 2. Materials and Methods

### 2.1. Cells and Reagents

Human immortalized keratinocytes (HaCaT) were purchased from Cell Lines Service GmbH (Eppelheim, Germany). Recombinant human TNF-α was purchased from PeproTech (Rocky Hill, NJ, USA). Oxyresveratrol (OXY) was purchased from Sigma-Aldrich (Saint Louis, MO, USA). MTT reagent (3-(4,5-dimethylthiazol-2-yl)-2,5-diphenyltetrazolium bromide) was purchased from Sigma-Aldrich (Saint Louis, MO, USA). Guava^®^ Cell Cycle Reagent was purchased from Luminex Corporate (Austin, TX, USA). Rabbit anti-phospho-AKT (Ser473) antibody, mouse anti-AKT antibody, rabbit anti-phospho-GSK3-_β_ (Ser9), mouse anti-GSK3-_β_ antibody, rabbit anti-Ki-67 antibody, rabbit anti-MCL-1 antibody, DAPI (4′, 6-diamidino-2-phenylindole, dihydrochloride), and LY294002 (a specific inhibitor of the PI3K/AKT) were purchased from Cell Signaling Technology (Boston, MA, USA). Goat anti-mouse IgG-IRDye^®^800CW and goat anti-rabbit IgG-IRDye^®^680RT were purchased from Li-COR Biosciences (Lincoln, NE, USA). Goat anti-rabbit conjugated with Alexa488 was purchased from Thermo Fisher Scientific (Waltham (HQ), MA, USA).

### 2.2. Cell Culture and TNF-α Treatment

HaCaT cells were cultured with Dulbecco’s modified Eagle’s media (DMEM) (Thermo Fisher Scientific, Waltham, MA, USA), containing 10% fetal bovine serum (Merck KGaA, Darmstadt, Germany), 100 U/mL penicillin, and 100 μg/mL streptomycin (both antibiotics from Thermo Fisher Scientific, Waltham, MA, USA), and incubated in an incubator set for proper humidity, temperature at 37 °C, and CO_2_ at 5%. The medium was changed every three days until the culture reached approximately 70% confluent. Cells were subcultured when they were 90% confluence. To determine the effect of TNF-α on cell number and cell cycle progress of HaCaT cells, different final concentrations (20–100 ng/mL) of TNF-α in DMEM, without fetal bovine serum, were added to HaCaT cells for 48 h. For other experiments, the highest concentration at 100 ng/mL of TNF-α was utilized.

### 2.3. MTT Assay

HaCaT cells at 1 × 10^4^ cells/well in DMEM were seeded in 96-well plates and incubated for 24 h. Then, cells were treated with varied concentrations of oxyresveratrol (OXY) with or without the existence of 100 ng/mL of TNF-α for 48 h. Cells were washed once with 1× PBS and incubated with 200 μL of the medium containing MTT reagent at the final concentration of 0.5 mg/mL for 1 h. Next, the MTT reagent was discarded, and each well was washed once with 1× PBS before adding 100 μL of 100% DMSO. Colorimetric measurement at 570 nm was performed using a microplate reader (BioTek Instruments, Winuski, VT, USA).

### 2.4. Cell Counting

HaCaT cells at a density of 4 × 10^5^ cells/well in DMEM were seeded in 24-well plates and cultured for 24 h. The medium was replaced with FBS-free medium, and the cells were treated with OXY at different concentrations (0, 10, 20, and 40 µM) with or without the presence of 100 ng/mL of TNF-α. Incubation time was designed to cover 3 days; cells were collected and counted at 0, 24, 48, and 96 h. Cell collection was performed by washing cells with 1× PBS and treating cells with 1× trypsin solution (100 µL) for 10 min before adding 400 µL of complete medium (DMEM with FBS). Cell suspension in each well was counted for three times by using the CellDrop™ Automated Cell Counter (DeNovix Inc., Wilmington, DE, USA).

### 2.5. Western Blot Analysis

HaCaT cells in DMEM were seeded in 3-cm dishes a density of 0.5 × 10^6^ cells/well and cultured for 24 h. The medium was replaced by adding 2 mL of FBS-free medium, and cells were cultured for 24 h. Then, cells were treated with OXY at 40 µM for 3 h, before stimulating with 100 ng/mL of TNF-α. Cell lysates were collected at different time points (0, 10, 20, 30, 40, 40, 60, 90, 120, 150, 180, and 210 min) by adding 300 µL of 1× reducing Laemmli buffer. Next, the cell lysates were heated at 95 °C for 5 min, separated by SDS-PAGE using 10% gel, and transferred onto polyvinylidene difluoride (PVDF) membranes (GE Healthcare Life Science, Marlborough, MA, USA). After blocking with blocking solution (5% bovine serum albumin (BSA) in TBST) for 1 h at room temperature (RT), membranes were incubated overnight with 1:1000 (diluted in blocking solution) of antibodies against phosphorylated Ser473 of AKT, total AKT, phosphorylated Ser9 of GSK3-_β_, and total GSK3-_β_. Membranes were washed trice with TBST (5 min each time), then membranes were incubated with proper secondary antibodies for 1 h. After washing membranes for 5 times with TBST, immunoreactive bands were visualized and recorded with the Odyssey^®^ CLx Imaging System (LI−COR Biosciences, Lincoln, NE, USA). The band intensity was analyzed and quantified using the ImageJ software. For additional experiments to assay a dose-dependent effect of OXY, we performed similar procedures, but the difference was that we varied concentrations of OXY; 10, 20, and 40 µM were examined. Moreover, an inhibitor of PI3K, LY294002, at 10 µM was included. Based on the results from Western blot where it showed that phosphorylation of AKT (pAKT) and GSK3-_β_ (p-GSK3-_β_) was peaked at 40, and 20 min after TNF-α stimulation, respectively, the harvesting time point for detecting pAKT was performed at 40 min after TNF-α stimulation, whereas the time for detecting GSK3-_β_ was at selected at 20 min post- TNF-α stimulation.

### 2.6. Immunofluorescence Study

HaCaT cells at a density of 0.5 × 10^6^ cells/well in DMEM were seeded onto glass coverslips placed in 3-cm dishes and cultured for 24 h. The medium was then replaced by FBS-free DMEM, and cells were cultured for 24 h. Cells were pre-treated with OXY at 40 µM for 3 h before stimulating with TNF-α for 40 min (for pAKT staining) and 20 min (for p-GSK3-_β_ staining). For Ki-67 and MCL-1, the OXY-treated cells were stimulated with TNF-α for 24 h. Cells were washed once with 1× PBS and fixed with 4% paraformaldehyde for 15 min. Sample coverslips were washed 3 times with PBS (5 min each time) and permeabilized with 0.3% TritonX-100 in PBS for 5 min. After washing trice, sample coverslips were blocked with 1% BSA in PBS for 1 h at RT, and incubated with primary antibodies in a moist chamber at 4 °C overnight. Sample coverslips were washed 3 times with 1× PBS and incubated with a secondary antibody conjugated with Alexa488 for 2 h in a moist chamber at RT. Sample coverslips were washed 3 times with 1× PBS and 1 time with deionized water (5 min) before mounting with Fluoromount-G (SouthernBiotech, Birmingham, AL, USA). Signals of the target proteins were visualized at 100× magnification by a fluorescent microscope, Axio Vert.A1 (Carl Zeiss, Jena, Germany). The Zen 2.6 (blue edition) software for the Zeiss Axiocam 506 color microscope camera was used to capture and analyze the images.

### 2.7. Colony Forming Assay

HaCaT cells were seeded in 3-cm dishes in DMEM at a low density of 5 × 10^4^ cells/well. Cell suspension was gently added into each well to create single cells with homogeneous cell distribution and cultured for 24 h. The medium was changed to an FBS-free medium, and cells were pre-treated with various concentrations of OXY (0, 10, 20, and 40 µM) for 3 h. Cells were then stimulated with 100 ng/mL of TNF-α. The size of the HaCaT colony at 0, 24, 48, and 96 h post-treatment was monitored and captured by an Axio Vert.A1 microscope (Carl Zeiss, Germany) equipped with the Zen 2.6 (blue edition) Software for the Zeiss Axiocam 506 color microscope camera. In addition, cell counting was performed after 96 h to verify the association of the colony size and the cell number.

### 2.8. Cell Cycle Analysis by Flow Cytometry

Cells were seeded in 24-well plates at a density of 4 × 10^5^ cells/well in DMEM for 24 h. The medium was replaced by a serum-free medium, and cells were cultured for 24 h. Next, cells were pre-treated with OXY at different concentrations (0, 10, 20, and 40 µM) for 3 h and stimulated with 100 ng/mL of TNF-α for 48 h. Culture medium was discarded and cells were rinsed with 1× PBS for 3 times. Cells were collected by trypsinization for 10 min and washed once with 1× PBS by centrifugation. Cells were then fixed and permeabilized by 70% ice-cold ethanol in a dropwise fashion to prevent cell aggregation. Cells were fixed for 24 h at −20 °C overnight. Cells were washed with 1× PBS for 1 time by centrifugation, and 150 µL of Guava^®^ Cell Cycle Reagent was added to each group of cells and incubated in the dark for 15 min at RT. Stained cells were analyzed immediately by flow cytometry using a flow cytometer DxFLEX from Beckman Coulter (Indianapolis, IN, USA). Data analysis was performed using the CytExpert for DxFLEX software, and results were presented as the ratio of cell population in G1, S, and G2/M phases.

### 2.9. Statistical Analysis

Data are presented as mean ± standard error of mean (SEM). Differences between groups were analyzed by one-way analysis of variance (ANOVA) with Tukey’s post hoc multiple comparisons on RAW data reads. Significant differences compared with appropriate controls are denoted with asterisks, * *p* < 0.05. All experiments were repeated at least three times independently.

## 3. Results

### 3.1. TNF-α Stimulates an Increase in Keratinocyte Cell Number

**Figure 1:**
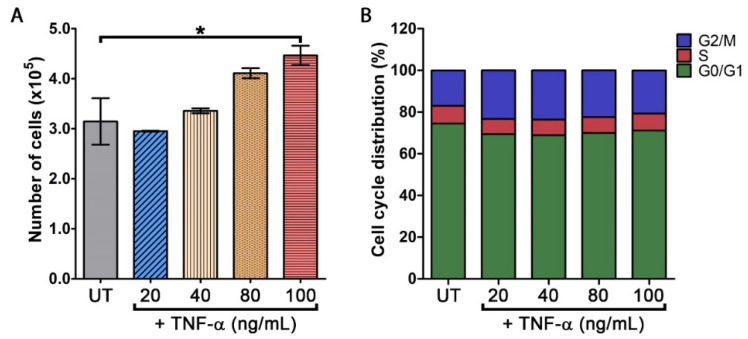
Effects of TNF-α on the proliferation and cell cycle distribution of human keratinocyte, HaCaT. (**A**) Number of HaCaT cells treated with different concentrations of TNF-α (20, 40, 80, and 100 ng/mL) (UT = untreated cells) for 48 h assayed by direct cell counting. (**B**) Cell cycle distribution of TNF-α-stimulated HaCaT cells at 48 h. * *p* < 0.05 in comparison to the untreated group.

We first determined the effects of TNF-α on stimulating the proliferation of HaCaT cells. Cells treated with varied concentrations of TNF-α (0, 20, 40, 80, and 100 ng/mL) for 48 h were directly counted. Results showed that in comparison to the untreated HaCaT cells where the cell count was 3.14 ± 0.46 × 10^5^ cells/mL, TNF-α at 20 ng/mL did not create any significant change (2.95 ± 0.01 × 10^5^ cells/mL) (Figure 1A). However, TNF-α at 40, 80, and 100 ng/mL significantly increased the number of cells to 3.36 ± 0.05 × 10^5^, 4.11 ± 0.10 × 10^5^, and 4.46 ± 0.195 × 10^5^ cells/mL, respectively (Figure 1A). This dose-dependent increase in cell number in response to TNF-α at 40, 80, and 100 ng/mL was approximately equal to 6.8%, 30.68%, and 41.97%. Nevertheless, TNF-α at all concentrations did not have any effect on cell cycle distribution tested by cell cycle analysis as the percentage of the cell in the G1, S, and the G2/M phase of all TNF-α-treated groups was not different (Figure 1B). Since TNF-α at 100 ng/mL provided the strongest ability in stimulating HaCaT cell growth, we then selected TNF-α at this concentration for all experiments studying the potential inhibitory effects of oxyresveratrol.

### 3.2. Oxyresveratrol (OXY) Inhibits TNF-α-Induced Keratinocyte Proliferation

**Figure 2:**
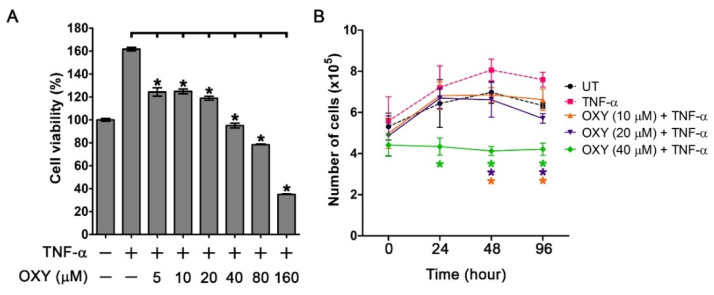
Effects of oxyresveratrol (OXY) on cell viability and proliferation of TNF-α-stimulated HaCaT cells. (**A**) Cell viability assay by MTT of HaCaT cells pre-treated with OXY (5, 10, 20, 40, 80, and 160 µM) for 3 h and stimulated with 100 ng/mL of TNF-α for 48 h. (**B**) Cell counting assay for quantifying the number of TNF-α-stimulated HaCaT cells treated with different concentrations of OXY (10, 20, and 40 µM) at various time points (0, 24, 48, and 96 h). * *p* < 0.05 in comparison to the TNF-α-treated group.

We further evaluated whether OXY has inhibitory effects against the proliferative influence of TNF-α. We first performed an MTT assay to examine the viability (which indirectly reflects proliferation) of HaCaT cells treated with various concentrations of OXY, ranging from 0 to 160 µM, in combination with 100 ng/mL TNF-α. We found that HaCaT cells treated with TNF-α alone exhibited a significant increase in cell viability to 160% compared to the untreated group (Figure 2A). Interestingly, OXY at 5, 10, 20, 40, 80, and 160 µM could suppress TNF-α-induced cell proliferation in a dose-dependent fashion (Figure 2A). To support these findings that OXY can suppress the proliferative ability of TNF-α, we performed a direct counting assay for analyzing the changes in cell number over the course of 96 h. Results revealed that TNF-α significantly increased HaCaT cell number over the course of 96 h, and the maximal number of cells was reached at 48 h post-treatment before the cell number declined at 96 h post-treatment (Figure 2B). As expected, OXY could potentially reduce HaCaT cell number over time, and the effect was dependent on the concentration of OXY. The strongest suppressive effects of OXY was clearly observed in the TNF-α-induced group treated with OXY at 40 µM where the cell number at all time points was stabilized to be approximately equal to that of the original seeding number at 0 h (Figure 2B).

### 3.3. OXY Blocks TNF-α-Induced Phosporylation of AKT and GSK3-_β_

**Figure 3:**
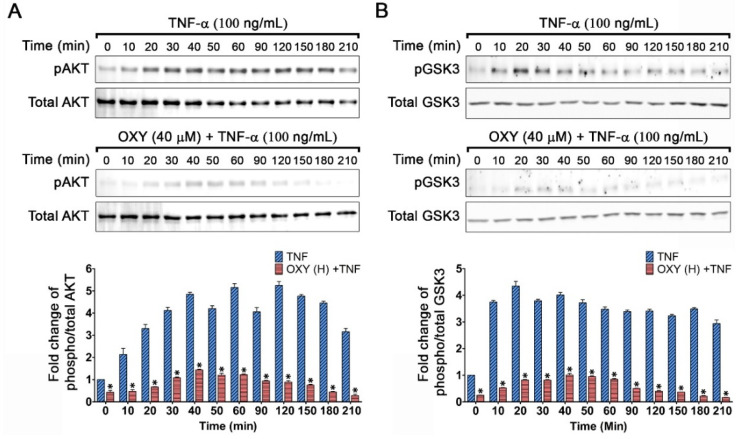
Inhibitory effects of OXY on AKT activation induced by TNF-α. (**A**) Phosphorylation status and quantitative analysis of AKT (pAKT) of HaCaT cells treated with OXY at the highest non-toxic concentration (OXY(H)) which was 40 µM and stimulated with 100 ng/mL TNF-α at various time points (0–210 min). (**B**) Phosphorylation status and quantitative analysis of GSK3-_β_ (GSK3-_β_) of HaCaT cells treated with OXY at the highest non-toxic concentration (OXY(H)) which was 40 µM and stimulated with 100 ng/mL TNF-α at various time points (0–210 min). * *p* < 0.05 in comparison to the TNF-α-treated group.

Since it is known that the PI3K/AKT is an important signaling pathway that can be stimulated by growth factors and cytokines including TNF-α, we next explored whether OXY can inhibit the activation of this pathway. We performed Western blot analysis by detecting the phosphorylation status of AKT and its downstream target, GSK3-_β_. When the cells were stimulated with TNF-α, phosphorylation of AKT was rapidly started at 10 min and gradually increased over the course of 210 min (Figure 3A). The fold change of phosphorylated AKT (pAKT) in TNF-α-stimulated cells harvested at 10, 20, 30, 40, 50, 60, 90, 120, 150, 180, and 210 min was 2.13 ± 0.27, 3.30 ± 0.18, 4.12 ± 0.14, 4.84 ± 0.09, 4.20 ± 0.12, 5.15 ± 0.16, 4.06 ± 0.19, 5.25 ± 0.17, 4.76 ± 0.75, 4.46 ± 0.44, and 3.16 ± 0.27 fold, respectively (Figure 3A). In contrast, cells stimulated with TNF-α with the presence of 40 μM of OXY strongly suppressed AKT phosphorylation at all time points (Figure 3A). The reduced level of pAKT by the action of OXY at 10, 20, 30, 40, 50, 60, 90, 120, 150, 180, and 210 min was 0.47 ± 0.07, 0.67 ± 0.01, 1.08 ± 0.04, 1.44 ± 0.03, 1.19 ± 0.08, 1.23 ± 0.02, 0.94 ± 0.02, 0.89 ± 0.02, 0.75 ± 0.01, 0.45 ± 0.01, and 0.27 ± 0.02 fold, respectively. Similar to pAKT, TNF-α rapidly induced phosphorylation of GSK3-_β_, and the phosphorylation status remained high (approximately 3 fold compared to that of the untreated group) throughout the course of 210 min (Figure 3B). However, the presence of OXY at 40 μM effectively blocked the kinase phosphorylation. Specifically, the cells harvested at all different time points exhibited the fold of phosphorylation around that of the untreated or lower (Figure 3B).

**Figure 4:**
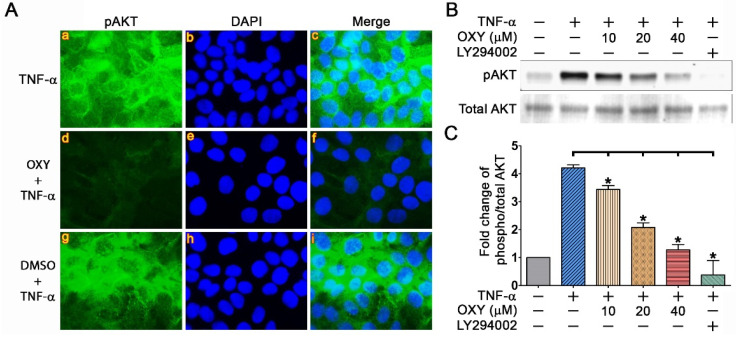
Effects of OXY on blocking TNF-α-stimulated AKT phosphorylation. (**A**) Immunofluorescence study detecting pAKT (green) in individual cells treated with 40 µM for 3 h and stimulated with 100 ng/mL TNF-α for 40 min. Nuclei were counterstained with DAPI (blue), visualization was performed at 100× magnification. (**B**) Western blot analysis showing dose-dependent effects of OXY on suppressing AKT phosphorylation. LY294002, a PI3K inhibitor, was used as a positive control. (**C**) Densitometric quantification of pAKT immunoreactive bands. * *p* < 0.05 in comparison to the TNF-α-treated group.

To verify the effects of OXY on blocking AKT phosphorylation, we performed an immunofluorescence study to visualize the localization pattern and the intensity of the phosphorylation signal in individual cells. In consistent with the results from Western blot analysis, stimulating HaCaT cells with TNF-α for 40 min could strongly activate AKT phosphorylation (Figure 4A(a–c)). The cellular staining pattern indicated that pAKT resides throughout the cell with its concentration at the cell-to-cell contact. Undoubtedly, the pAKT signal dramatically decreased in the TNF-α-stimulated HaCaT cells treated with 40 μM of OXY (Figure 4A(d–f)). However, DMSO, which was used as a vehicle control, showed the positive signal intensity and staining pattern similar to those of the TNF-α-treated group (Figure 4A(g–i)). We further confirmed our findings by testing the effect of OXY at lower concentrations and using Western blot analysis. Data clearly indicated that OXY could effectively suppress AKT phosphorylation in a concentration-dependent manner (Figure 4B). An inhibitor of PI3K, LY294002, which can inhibit phosphorylation of AKT, was also included in this experiment as a positive control, and this inhibitor at 10 μM could potently block AKT phosphorylation (Figure 4B). Quantitative analysis showed that TNF-α increased AKT phosphorylation to about 4 fold (Figure 4C). OXY at 10, 20, and 40 μM significantly inhibited TNF-α-stimulated AKT phosphorylation to approximately 3.5, 2, and 1.5 fold, respectively, while LY294002 at 10 µM reduced pAKT to around 0.5 fold (Figure 4C).

**Figure 5:**
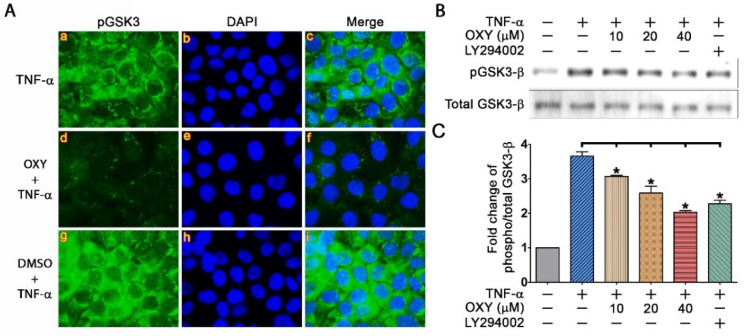
Effects of OXY on blocking TNF-α-stimulated GSK3-_β_ phosphorylation. (**A**) Immunofluorescence study detecting pGSK3-_β_ (green) in individual cells treated with 40 µM for 3 h and stimulated with 100 ng/mL TNF-α for 20 min. Nuclei were counterstained with DAPI (blue), visualization was performed at 100× magnification. (**B**) Western blot analysis showing dose-dependent effects of OXY on suppressing GSK3-_β_ phosphorylation. LY294002, a PI3K inhibitor, was used as a positive control. (**C**) Densitometric quantification of pGSK3-_β_ immunoreactive bands. * *p* < 0.05 in comparison to the TNF-α-treated group.

Consistent with pAKT, detection of GSK3-_β_ by immunofluorescence study revealed a strong signal of pGSK3-_β_ in the cytoplasm of TNF-α-stimulated HaCaT cells (Figure 5A(a–c)), and OXY at 40 μM clearly diminished the phosphorylation status of this kinase (Figure 5A(d–f)). Moreover, Western blot analysis confirmed an inhibitory effect of OXY on TNF-α-stimulated GSK3-_β_ phosphorylation in a concentration dependent fashion (Figure 5B,C).

### 3.4. OXY Suppresses TNF-α-Induced HaCaT Colony Expansion and Causes Cell Cycle Arrest

**Figure 6:**
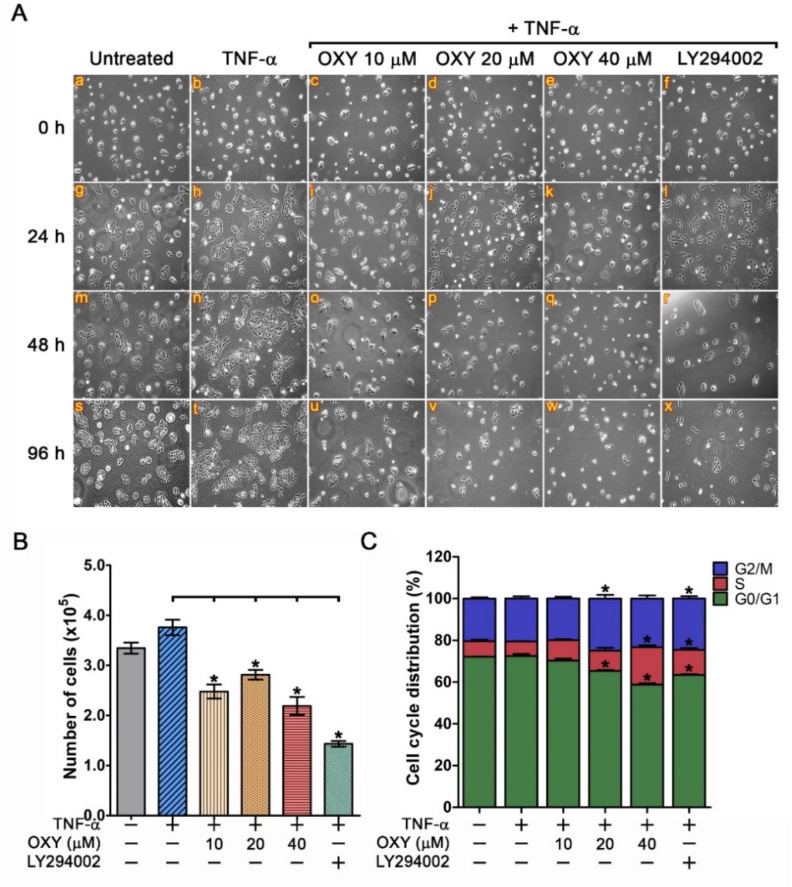
Effects of OXY on TNF-α-induced HaCaT cell proliferation and cycle progress. (**A**) Colony forming assay of HaCaT cells pre-treated with OXY extract at different concentrations (10, 20, and 40 µM) for 3 h, stimulated with 100 ng/mL of TNF-α for, and observed by a phase-contrast microscope (10× magnification) at different time points (0, 24, 48, and 96 h). (**B**) Cell counting assay for TNF-α-stimulated HaCaT cells treated with OXY at different concentrations and at different time points. (**C**) Cell cycle analysis indicating the effects of OXY on cell cycle distribution. * *p* < 0.05 in comparison to the TNF-α-treated group.

**Figure 7:**
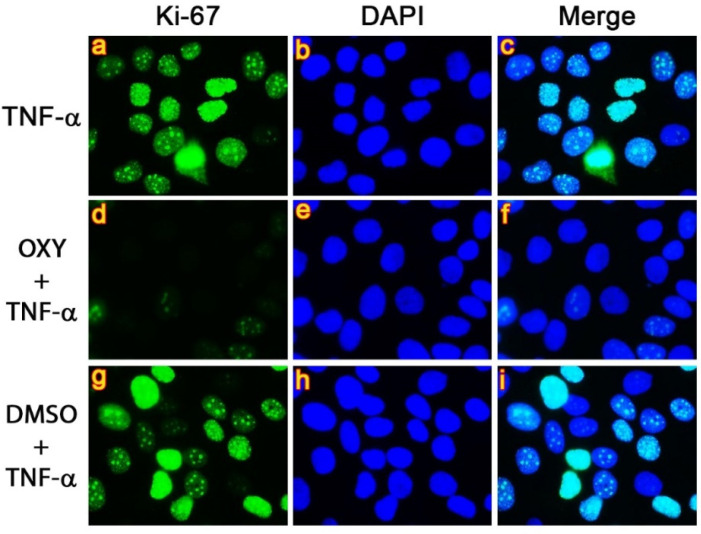
Inhibitory effects of OXY on TNF-α-stimulated expression of Ki-67 (green) in the nuclei of HaCaT cells. Nuclei were stained with DAPI (blue). Visualization was performed at 100× magnification. Picture (**a**–**c**) present TNF-α-stimulated cells showing Ki-67 staining, DAPI staining, and the merged images, respectively. Picture (**d**–**f**) represent OXY-treated cells stimulated with TNF-α showing Ki-67 staining, DAPI staining, and the merged images, respectively. Picture (**g**–**i**) represent DMSO-treated cells stimulated with TNF-α showing Ki-67 staining, DAPI staining, and the merged images, respectively.

The observation that TNF-α-stimulated AKT and GSK3-_β_ phosphorylation was strongly inhibited by OXY led to the hypothesis that OXY may inhibit the formation of the HaCaT colony. Therefore, we performed a colony forming assay and found that in comparison to the untreated group, TNF-α clearly induced the size of the cell colony, but all OXY at all concentrations and LY294002 could effectively inhibit the action of TNF-α (Figure 6A). Consistently, when we directly counted and quantified the number of cells in each group, OXY at all concentrations and LY294002 significantly reduced the HaCaT cell number under the influence of TNF-α (Figure 6B). We further investigated the anti-proliferative action of OXY by cell cycle analysis. Results showed that the untreated cells and TNF-α-stimulated cells exhibited similar pattern of the cell cycle distribution (Figure 7c). However, treatment of TNF-α-stimulated cells with OXY (especially at 40 µM) presented a significant trend of reduction in the G1-phase cell population, but an increase in the S-phase or G2/M-phase cell population (Figure 6C). Similarly, LY294002 caused G1-phase cell reduction and induced cell cycle arrest at S and G2/M phases (Figure 6C).

### 3.5. OXY Inhibits TNF-α-Induced Expression of Proliferation and Survival Markers in HaCaT Cells

Since we observed that OXY blocks phosphorylation of AKT and GSK3-_β_, which caused cell cycle arrest and significant reduction in number of cells stimulated with TNF-α, we performed an immunofluorescence study to confirm these results by detecting a proliferative marker (Ki-67) and an anti-apoptotic protein (MCL-1), which is one of the cell survival markers. Data from immunofluorescence study obviously presented high expression of Ki-67 in the nuclei of TNF-α-stimulated cells (Figure 7a–c), and similar observation was seen in the group of DMSO vehicle control (Figure 7g–i). However, OXY at 40 µM intensely suppressed the nuclear expression of Ki-67 (Figure 7d–f).

**Figure 8:**
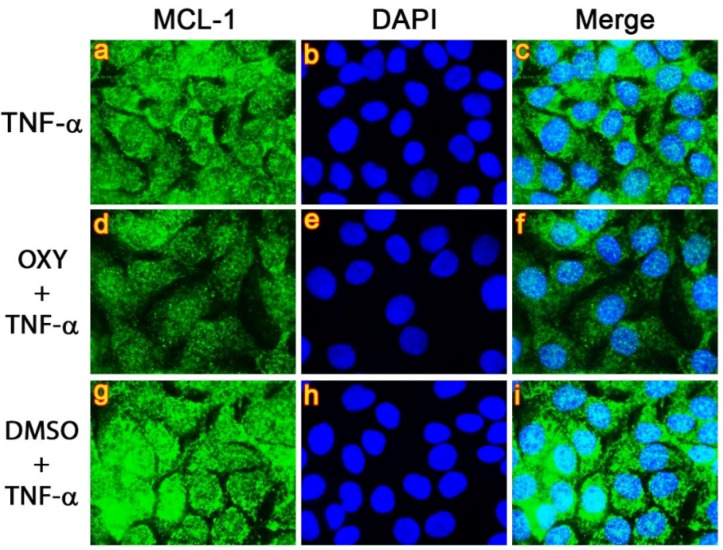
Inhibitory effects of OXY on TNF-α-stimulated expression of MCL-1 (green) in HaCaT cells. Nuclei were stained with DAPI (blue). Visualization was performed at 100× magnification. Picture (**a**–**c**) present TNF-α-stimulated cells showing MCL-1 staining, DAPI staining, and the merged images, respectively. Picture (**d**–**f**) represent OXY-treated cells stimulated with TNF-α showing MCL-1 staining, DAPI staining, and the merged images, respectively. Picture (**g**–**i**) represent DMSO-treated cells stimulated with TNF-α showing MCL-1 staining, DAPI staining, and the merged images, respectively.

Similarly, the cytoplasmic signal of MCL-1 in the OXY-treated group (Figure 8d–f) was much weaker than those in the TNF-α-stimulated cells (Figure 8a–c) and DMSO vehicle control (Figure 8g–i).

## 4. Discussion

Medicinal plants have been reported to alleviate TNF-α-induced HaCaT cell proliferation [44,45]. However, the direct inhibitory effects on the PI3K/AKT signaling pathway in response to TNF-α stimulation have not been reported. Previously, we discovered that oxyresveratrol (OXY) has strong anti-inflammatory activity, in part through inhibiting LPS-induced activation of AKT in mouse macrophage cells [38] and IL-1β induced AKT in human microglia [39]. These reports led us to believe that OXY may play a conserved role in suppressing AKT activation in different cell types and different stimulants. To pace into our ultimate goal for developing OXY as a potential treating agent for psoriasis, we therefore focused mainly on exploring the anti-proliferative effects of OXY against TNF-α stimulation in keratinocyte, using HaCaT as a study model.

Data from our current study support that TNF-α potently stimulates HaCaT cell proliferation in a concentration dependent manner, suggesting that the presence of TNF-α at the skin lesion from the invading immune cells may be one of the key players contributing to an increase in the number of keratinocyte. These data were consistent with studies from other groups reporting this similar proliferative activity of TNF-α in the same cell line [45] and in different cell types [17]. However, our cell cycle analysis results did not indicate any aberration in the cell cycle distribution of HaCaT cells stimulated with TNF-α, indicating that TNF-α accelerates cell proliferation via normal cell cycle control. To evaluate the anti-proliferative effect of OXY, we first performed cell viability assay and found that OXY showed the reduction trend of cellular metabolic activity in a dose-dependent fashion. This data suggest that OXY interferes with the effect of TNF-α on increasing cell viability, and this observed event may be caused by the suppression of HaCaT cell proliferation. We then performed an experiment to directly count the cells to ensure our hypothesis. As expected, the numbers of TNF-α-stimulated HaCaT cells with the presence of various concentrations of OXY was significantly lower than those of the cells stimulated with TNF-α alone at all-time points over the course of 96 h. In particular, the highest concentration of OXY, which was 40 µM, was able to maintain the cell number close to that of its original seeding density at 0 h. These results reflect the strong inhibitory activity of OXY on keratinocyte cell proliferation under the influence of TNF-α.

The observation that TNF-α stimulated HaCaT cell proliferation directs us to realize the role of the PI3K/AKT signaling pathway since this particular signaling pathway creates an intracellular network for the regulation of cell proliferation [46]. Specifically, extensive studies have revealed the high expression level of PI3K and AKT as well as the accumulation of phosphorylated AKT (pAKT) in the keratinocyte in psoriasis lesions in comparison to normal and non-lesional skin [10,11]. Therefore, we stimulated HaCaT cells with TNF-α (with and without OXY treatment) and detected the phosphorylation status of AKT and one of its downstream targets, GSK3-_β_, at different time points. Data clearly showed that OXY could potentially suppress the TNF-α-induced activation of AKT and GSK3-_β_. Consistently, when we performed an immunofluorescence study, OXY also exhibited its potency in blocking the activation of AKT and GSK3-_β_ individual cells upon TNF-α stimulation. Similarly, Western blot analysis further confirmed that OXY could truly inhibit the activation of the two kinases in a dose-dependent manner. The highest concentration of OXY showed the inhibitory pattern quite similar to that of a PI3K inhibitor (LY294002), verifying that OXY authentically blocks the PI3K/AKT signaling pathway in response to TNF-α induction. We further investigated, by performing functional tests, to understand how OXY interferes the proliferative effect of TNF-α. A colony expansion assay clearly showed that OXY blocked the colony forming of HaCaT cells, and this was related to the reduced number of cells over the period of 96 h. Moreover, we finally discovered that OXY significantly induced cell cycle arrest at S and G2/M phases in a pattern similar to that of the PI3K inhibitor. It is reasonable to obtain this phenomena as a result of suppressing the TNF-α-induced PI3K/AKT signaling pathway by OXY since it is well defined that phosphorylated AKT controls the intracellular functions of its substrates related to cell cycle progression at the G1/S and G2/M transitions [47]. To be more confident, we stained for the expression of Ki-67 in the nuclei since this protein is a proliferative marker [47]. As anticipated, OXY drastically decreased the expression of Ki-67 in the nuclei of TNF-α-stimulated HaCaT cells, and the reduction of Ki-67 expression supports the findings from our cell cycle analysis. Therefore, these results clearly explain that a decrease in TNF-α-stimulated cell proliferation is likely the consequence of suppression of the activation of the PI3K/AKT signaling pathway by OXY. Additionally, since PI3K/AKT signaling is also responsible for cell survival, this implies that OXY may down regulate the expression of an anti-apoptotic protein, MCL-1, which is one of major downstream targets of AKT activation. Our result demonstrated that the intracellular level of MCL-1 in individual cells was dramatically reduced by the action of OXY. This confirms our hypothesis that OXY can also manipulate the effect of TNF-α on keratinocyte. Notably, to fully understand how OXY interacts with the target kinases, future structural biology studies [48] or rational redesign of orthogonal kinase-substrate pairs to design synthetic signaling pathways [49] should be conducted.

**Figure 9:**
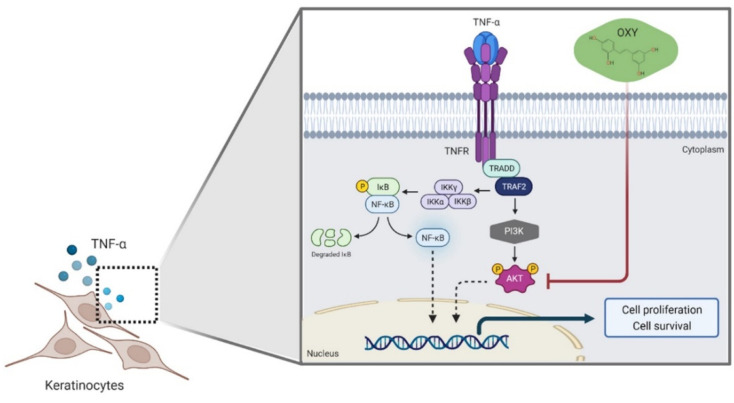
Illustrative picture presenting that OXY regulates TNF-α-induced immune response by inhibiting the activation of the PI3K/AKT signaling cascade, leading to a decrease in cell proliferation and survival. The graphic was created with BioRender.com (accessed on 25 November 2021).

On the basis that during the development of psoriasis, responsible immune cells (T cells) extensively secrete TNF-α, which then play a significant role in the pathogenic process [5], there has been an attempt to develop TNF-α inhibitors as an optional therapy for psoriasis. In particular, certain TNF-α inhibitors were proved to be highly effective and well-tolerated in plaque psoriasis in long-term observation [50,51]. Nevertheless, since a series of stimulating factors contribute to the pathophysiology of psoriasis, the treatment of this disease is therefore complicated. One major finding is that the PI3K/AKT pathway is aberrantly stimulated in hyperproliferation keratinocyte, making this specific pathway a potential therapeutic target of psoriasis. In view of the vital function of the PI3K/AKT signaling pathway in regulating keratinocyte proliferation, development of therapeutic drugs using PI3K and AKT inhibitors is of great significance for the treatment of psoriasis [52]. Considering the fact that OXY is an active natural compound that exhibits potent inhibition of the PI3K/AKT signal transduction pathway and proliferation induced by TNF-α (as depicted in Figure 9), our discovery sheds light on the possibility to develop OXY as a novel PI3K/AKT-based therapy to improve psoriasis treatment effectiveness. To ensure our belief, research investigation in psoriasis-induced animal models and in patients with psoriasis should be performed to provide definitive evidence for the efficacy of OXY monotherapy in curing psoriasis. Moreover, clinical studies that focus on combinatorial effects of OXY on suppressing TNF-α-stimulated PI3K/AKT and other currently available drugs with different mechanisms of action should be conducted.

## Data Availability

The data presented in this study are available in this article.
